# Melatonin and cancer suppression: insights into its effects on DNA methylation

**DOI:** 10.1186/s11658-022-00375-z

**Published:** 2022-09-05

**Authors:** Amirhossein Davoodvandi, Banafsheh Nikfar, Russel J. Reiter, Zatollah Asemi

**Affiliations:** 1 Student Research Committee, Kashan University of Medical Sciences, Kashan, Iran; 2 Cancer Immunology Project (CIP), Universal Scientific Education and Research Network (USERN), Tehran, Iran; 3 Pars Advanced and Minimally Invasive Medical Manners Research Center, Pars Hospital, Iran University of Medical Sciences, Tehran, Iran; 4 Department of Cell Systems and Anatomy, UT Health. Long School of Medicine, San Antonio, TX USA; 5 Research Center for Biochemistry and Nutrition in Metabolic Diseases, Institute for Basic Sciences, Kashan University of Medical Sciences, Kashan, Iran

**Keywords:** Melatonin, DNA methylation, DNMT, Epigenetics

## Abstract

Melatonin is an important naturally occurring hormone in mammals. Melatonin-mediated biological effects include the regulation of circadian rhythms, which is important for optimal human health. Also, melatonin has a broad range of immunoenhancing actions. Moreover, its oncostatic properties, especially regarding breast cancer, involve a variety cancer-inhibitory processes and are well documented. Due to their promising effects on the prognosis of cancer patients, anti-cancer drugs with epigenetic actions have attracted a significant amount of attention in recent years. Epigenetic modifications of cancers are categorized into three major processes including non-coding RNAs, histone modification, and DNA methylation. Hence, the modification of the latter epigenetic event is currently considered an effective strategy for treatment of cancer patients. Thereby, this report summarizes the available evidence that investigated melatonin-induced effects in altering the status of DNA methylation in different cancer cells and models, e.g., malignant glioma and breast carcinoma. Also, we discuss the role of artificial light at night (ALAN)-mediated inhibitory effects on melatonin secretion and subsequent impact on global DNA methylation of cancer cells.

## Melatonin and its role in human health

Melatonin is an endogenous metabolic hormone that has an obvious association with the regulation of circadian rhythms. In addition to its effect in sleep promotion, melatonin has many other functions including its role in neutralizing reactive oxygen species (ROSs), identified as its antioxidant properties, immunomodulation and inflammatory disorders, and anti-viral actions [1–3]. Moreover, the evidence is compelling that melatonin has a variety of anti-cancer effects, such as its inhibition of cancer cell viability, proliferation, progression, and metastasis or even inhibition of cancer initiation [3, 4]. This has drawn attention to the potential use of melatonin for cancer treatment in the clinical setting, although huge obstacles still exist before its wide clinical administration is accepted [5].

### Melatonin biosynthesis

Melatonin (N-acetyl-5-methoxy-tryptamine) is an endogenous hormone was discovered in bovine pineal tissue of bovine by Lerner and colleagues in 1958 [6]. Later, melatonin was reported to be existed in different plants, unicellular organisms such as bacteria, and clades of invertebrates [7–9]. The pineal gland releases melatonin into the third ventricle and into the blood. Furthermore, the biosynthesis of melatonin occurs in bone marrow and lymphocytes, gastrointestinal (GI) tract, and eyes, etc., and perhaps in every cell [10]. Melatonin biosynthesis take places in the mitochondria of eukaryotic cells [11]. Initially, cells take up the essential circulating amino acid, tryptophan, and then convert it to 5-hydroxytryptophan, and serotonin in two consecutive reactions catalyzed by tryptophan hydroxylase, and 5-hydroxytryptophan decarboxylase, respectively. After a reaction catalyzed by aralkylamine N-acetyltransferase (AANAT), serotonin transformed into N-acetyl-5-hydroxytryptamine, succeed by its methylation to melatonin by acetylserotonin-O-methyltransferase [12].

The “master biological clock”, located in the suprachiasmatic nucleus (SCN) of the hypothalamus, has essential regulatory actions in determining the rhythmic production of pineal melatonin; this master circadian regulator is present in the brain of all mammals including the human [13]. Photoreceptor cells of retina respond to light information with the neural signal being transferred to the hypothalamic SCN through the retinohypothalamic tract located in the optic nerve. The SCN by means of efferent neurons sends this information via intermediolateral column to superior cervical ganglia (SCG), with the postganglionic sympathetic fibers then projecting to the pineal gland to suppress melatonin synthesis. In the absence of light at night, sympathetic neurons ending in the pineal release noradrenaline which leads to the AANAT activation, and subsequently increases the production and release of melatonin. After its release into the CSF and blood, melatonin exerts a plethora of biological effects as enumerated above.

### Melatonin has an extended range of biological activities

Melatonin possesses a wide spectrum of biological and physiological properties, including its effects on the regulation of circadian rhythms and its remarkable efficacy as an anti-oxidant [1]. In addition, studies have shown that the melatonin and immune system have a two-way association: the immune system has actions that promote melatonin biosynthesis and, conversely, melatonin modulates immune system responsivity [14]. Interestingly, melatonin also has potent anti-inflammatory properties via reducing the expression levels of tumor necrosis factor alpha (TNF-α), interferon-gamma (IFN-γ), and interleukin-2 (IL-2) and by up-regulating the expression of anti-inflammatory cytokines such as, IL-4, IL-10, and IL-27 [15]. A large number of investigations have reported promising melatonin-induced regulatory impacts on cancer management in numerous stages of this chronic disorder, such as cancer initiation, progression, and metastasis [16]. Accordingly, affecting distinct metastatic-related molecular pathways and cellular processes including matrix metalloproteinases (MMPs), Rho-associated kinase protein‐1, and epithelial-to-mesenchymal transition (EMT), melatonin has promising properties against metastasis of tumoral cells [17, 18].

These biological properties have a broad spectrum of molecular mechanisms, including binding to receptors of cell membranes, interacting with different proteins in cytosol and nucleus, and direct scavenging of free radicals such as ROSs [1]. Three different classes of putative receptors for melatonin have been characterized: in the membrane, MT1 and MT2 are members of the superfamily of G-protein-coupled receptors which are encoded by MTNR1A and MTNR1B, respectively, [19]; retinoid orphan receptors (RORs), located in the nucleus, belong to the steroid receptor superfamily; they have been shown to bind melatonin [20, 21]; finally, the MT3 melatonin binding site, also is known as quinone reductase 2 (QR2), is situated in the cytosol [22]. Melatonin-induced activation of either MT1 or MT2 receptors causes decreased activity of adenylyl cyclase and, subsequently, reduced levels of cyclic adenosine monophosphate (cAMP); this leads to protein kinase A (PKA) activity repression. Additionally, MT2 also interferes with the activation of guanylyl cyclase and the subsequent formation of cyclic guanosine monophosphate (cGMP) [23]. MT3 functions in detoxification and as an anti-oxidant enzyme and in the reduction of cell proliferation [24]. The membrane receptors for melatonin occur in the majority of cell types such as pituitary gland, brain, hypothalamic SCN, retinal, renal, pancreatic, fat, and immune cells [25, 26].

Melatonin’s widely different biological actions involve the induction distinct mechanisms. The precise melatonin application in numerous diseases and health problems could be facilitated by a thorough understanding of the identified mechanisms [27]. Melatonin structural modification based upon its different receptors, could be considered as an effective strategy for decreasing the specific melatonin-mediated effects.

## Epigenetic regulatory mechanisms

Histone modifications, non-coding RNAs, and DNA methylation are major epigenetic processes for altering transcription without inducing any changes in DNA sequence of mammalian cells. These processes are potent biological regulators of various cellular activities and are influenced by environmental agents including nutrition status, stress and infections, chemical substances and drugs [28, 29]. Studies have shown that alterations in epigenetic status are strongly associated with the incidence of neoplasms [30, 31], autoimmune diseases [32], obesity and cardiovascular diseases (CVDs) [33], and type 2 diabetes mellitus (T2DM) [34]. Therefore, identifying novel bioactive chemicals for modification of epigenetic processes, especially in patients with cancer, is considered an effective approach for establishing novel treatments for these patients. In this review, we discuss how melatonin affects epigenetic modifications with a special focus on its ability to influence DNA methylation.

### DNA methylation

DNA methylation dynamics are considered promising epigenetic signatures and many studies have extensively investigated in comparison with other epigenetic processes. In recent years, dynamic control of DNA methylation has been widely incorporated into modern epigenetic models. In mammals, DNA epigenetic modifications generally involve a methyl group (-CH3) addition to a cytosine nucleotide, with the subsequent generation of 5-methylcytosine (5mC). Controlling the DNA methylation is the principal function of a family of enzymes named DNA methyltransferases (DNMTs) [35]. In mammals, methylation of DNA frequently occurs in the CpG dinucleotide islands. CpG dinucleotide framework is presented as cytosine and guanine and a phosphate group between these two nucleotides. CpG islands are recognized as dense CpG dinucleotides frequently occurring in promoter and other regulatory regions such as in interspersed areas [36–39]. The number of CpG sites in the human genome is estimated to be about 29 million. Approximately 60–80% of these sites are methylated in human somatic cells [40]. It is clear that CpG sites do not have a uniform distribution pattern in the genome; conversely, a greater part of the genome is devoid of CpG sites [36, 41, 42]. Interestingly, methylation of DNA take places in 70% and 40% of all CpG dinucleotides, and CpG-rich island genes, respectively [43]. Different methyl-binding proteins recognize methylated CpG dinucleotides as binding sites participating in the recruitment of either protein or machinery of chromatin-remodeling, resulting in the facilitation of gene inactivation and silencing, and condensation of chromatin [44–46]. Methylation of DNA in non-CpG sites has also not been found to influence the structure or stability of chromatin, DNA and protein interactions, or gene regulation [47–51].

Aberrant methylation of DNA has been extensively reported to be involved in an extended range of cancer types, including hepatocellular carcinoma (HCC) [52], colorectal cancer (CRC) [53], Wilms tumor [54], breast carcinoma [55], ovarian [56], and bone-related cancers such as osteosarcoma [57]. Besides, emerging evidence evaluating omics technologies have demonstrated that extensive cancer-related differential exists in the methylation of DNA [58–60]. As well as, different mutations in DNMTs, differences in DNMTs expression levels, and dysregulated expression of ten-eleven translocation enzymes (TETs) are repeatedly reported in numerous cancer types, they all suggesting a substantial association between aberrant methylation of DNA and cancer incidence [61–64]. Many studies reported that epigenetic alterations over large chromatin regions in cancer diseases results in epigenetic instability, and subsequent gene expression alterations [65–69]. While epigenetic processes are crucial events for regulating cellular plasticity and stem cell reprogramming in the development of normal cells [70, 71], but disrupted epigenetic alterations such as aberrant DNA methylation in cancer cells can lead to heterogeneity of tumor cells, and subsequent poor prognosis in cancer patients [72–74].

## Melatonin influences DNA methylation in cancer cells

Glioblastoma is the most prevalent primary tumor of brain. Furthermore, glioblastoma is a highly aggressive and lethal type of cancer; the average of life expectancy is reported to be less than 1 year after it is diagnosed. Because of the difficulty with complete surgical resection and high resistance of glioblastoma to existing chemotherapy agents, treatment of these patients remains exceedingly complicated. Therefore, there is an urgent need for developing unconventional therapeutic strategies in the treatment of patients with malignant glioblastoma. In the past few years, different investigations have shown the existence of stem cell-like cells in solid tumors involving malignant glioblastomas [75]. Stem cell-like cells are important built-in multidrug-resistant and pluripotent cells that commonly develop drug resistance and continue to proliferative after a chemotherapy regimen. Accordingly, the therapeutic approaches that cannot eradicate the brain tumor stem cells (BTSCs) are expected to be unsuccessful. While these treatments may be successful for killing an appreciable number of tumor cells and causing a transitory regression, they fail to alter cancer relapse [76]. For the purpose of achieving a persistent long-lasting treatment of glioblastoma patients, it is a requirement to discover and develop novel therapeutic strategies for targeting both BTSCs and tumor bulk.

Martin and colleagues [77] investigated the properties of melatonin and its combination with chemotherapeutic agents on these multi-drug resistant brain tumor stem cells. Results showed that melatonin and chemotherapeutic drugs in combination induced a remarkable synergistic toxicity against BTSCs and malignant A172 glioma cells. Co-treatment with temozolomide as current agent for remission of malignant glioma with melatonin, significantly correlated with down-regulated expression of ABCG2/BCRP and subsequent inhibition of ABC transporter functions. In fact, melatonin significantly elevated DNA methylation of the promoter of ABCG2/BCRP; thus, the expression properties and function of ABCG2/BCRP were prohibited due to the preincubation melatonin with a DNA methyltransferase inhibitor (DNMTi). Hence, their findings highlight a potent association between the decrease of ABCG2/BCRP activities and the synergistic toxicity of melatonin and therapeutic agents. By inducing DNA methylation in proto-oncogenes, melatonin also could be considered as a promising chemical for defeating multi-drug resistance in malignant glioblastomas, and improving the effectiveness of current chemotherapeutic regimens.

The circadian production and release of melatonin is involved in the regulating periodic events [78]. A photoperiod-independent production of melatonin also typically occurs in the gut. Yet, the physiological roles of locally-produced melatonin in the GI tract is poorly understood. Mannino et al. [79] assessed the melatonin-mediated anti-inflammatory activities in an *in vitro* model of intestinal inflammation using IL‐1β‐stimulated inflamed intestinal epithelium in Caco‐2 colon cancer cells. Caco‐2 cells differentiated monolayers were preincubated with 1 nmol/L to 50 µmol/L melatonin and then treated with IL‐1β. Following treatment, DNA‐breakage, the status of global DNA methylation, and numerous inflammatory mediators, were assayed. For evaluating the role of membrane receptors of melatonin, they also co-treated differentiated monolayers with melatonin and luzindole, an antagonist of the MT1 and MT2 receptor. The results proved that melatonin at similar concentrations to those absorbed by the intestinal lumen following ingestion of dietary supplements taken for the sleep disorders, decreases the IL‐1β-mediated inflammatory responses. Anti‐inflammatory properties attenuated the levels of pro-inflammatory agents including IL‐6, IL‐8, cyclooxygenase-2 (COX‐2), nitric oxide (NO), and paracellular permeability. Moreover, melatonin-induced protection was also related to decreased activation of nuclear factor-κB (NF‐κB) and prevention of DNA demethylation. In summary, their findings revealed that melatonin, via affecting local physiological activities and DNA methylation, modulates inflammation in the GI tract suggesting a novel therapeutic opportunity for the management of GI-tract-associated chronic disorders such as inflammatory bowel disease (IBD) and also cancer.

In recent decades, the incidence of breast cancer has steadily increased worldwide; also, its incidence is greater in more highly developed countries [80]. Industrialization- and urbanization-associated environmental factors are believed to be potent agents in the etiology of breast cancer. Prolonged and repeated exposure to the artificial light-at-night (ALAN) at night is one of the most prevalent changes of modern life [81]. Emerging evidence has documented a consistent association between ALAN and breast cancer incidence. As an illustration, large epidemiological investigations suggest that extended periods of rotating night shifts in women is associated with a moderate increase in breast cancer risk [82]. Schwimmer et al.[83] hypothesized that ALAN exposure decreases the secretion of melatonin, resulting in the induction of epigenetic modifications and greater growth-rate in breast cancer tumors. They also specifically investigated the impact of exposure to ALAN and exogenous melatonin on the growth-rate of breast cancer tumors. Accordingly, induction of breast cancer in BALB/c short day-acclimated 4T1 breast cancer tumor-bearing mice resulted in greater tumor growth. Results also showed that ALAN-induced impact for increasing growth-rate of breast tumors was strongly reversed by melatonin supplementation and the reduction in tumor growth was accompanied by an induction of global DNA methylation.

In a similar study, via analyzing DNA methylation profiles, Lee and colleagues [84] further investigated melatonin-induced epigenetic alterations in MCF-7 breast cancer cells to achieve a new perspective into the potential mechanisms of melatonin-mediated anti-cancer properties. The candidate genes-associated methylation status and expression levels of mRNAs were confirmed by bisulfite PCR and real-time quantitative PCR (qPCR) in melatonin-exposed cells. This approach using 1 nM melatonin-treated MCF-7 breast cancer cells resulted in the detection of different cancer-related genes, including down-regulated EGR3 and POU4F2/Brn-3b oncogenic genes, and up-regulated GPC3 tumor suppressor gene (TSG). Their findings provided further insights into the melatonin-induced patterns of DNA methylation, and moreover suggest a potential mechanism of the melatonin-mediated anti-cancer properties for modification of aberrant DNA methylation in breast cancer cells.

Agbaria et al.[85] evaluated the impact of 1 × 30 min/midnight ALAN exposure either with or without nocturnal melatonin supplementation on epigenetic processes and inhibition of tumor development in 4T1-inoculated BALB/c mice. At the conclusion of the experiment, the tissues from lungs, liver, spleen and tumor were extracted to analyze the levels of DNMT total activity and global DNA methylation. ALAN-exposed mice showed significant reduction in the levels of 6-sulfatoxymelatonin and remarkable increase in tumor volume, body weight, and lung metastasis in comparison with controls in which all indices were diminished following melatonin supplementation. Compared to tissues from control animals, enzymatic activity and global DNA methylation levels were reported to be lower in breast tumor and liver tissues and higher in lungs and spleen following exposure to ALAN. Their finding proved that ALAN exposure results in the disruption of the melatonin rhythm, which leads to an increased breast cancer burden through influencing the activity of DNMT and global DNA methylation levels. These findings may have utility to be used for early detection and breast cancer management by monitoring melatonin levels and global DNA methylation.

Melatonin-induced inhibition of the chemoresistance of human breast cancer involves different mechanisms including tumor metabolism suppression, inhibition of different kinase enzymes, and transcription factors which usually are activated and involved in drug-resistant breast carcinoma [86]. Various studies have suggested that overexpressed levels of signal transducer and activator of transcription 3 (STAT3) is associated with chemoresistance to paclitaxel in breast cancer patients [87, 88]. Accordingly, STAT3 triggers DNMT1 expression for inducing epigenetic suppression of the transcription of Aplasia Ras homolog one (ARHI), which functions as a tumor suppressor and cytoplasmic STAT3 inhibitor for reversing paclitaxel resistance [89]. Xiang and colleagues [90] demonstrated that exposure of breast tumor–bearing rats to dim light at night (dLAN), and the consequential disrupted expression of the circadian melatonin rhythm resulted in the elevation of phosphorylated and acetylated STAT3 levels, up-regulated DNMT1, and down-regulated expression of ARHI and sirtuin 1 (SIRT1). Additionally, administration of either melatonin or its combination with SIRT1 significantly reversed IL-6-mediated STAT3 acetylation and ARH1 methylation for up-regulating mRNA expression level of ARH1 in MCF-7 cells. The results of this study demonstrated that dLAN-induced disruption of circadian melatonin cycle resulted in chemoresistance to paclitaxel through promoting the expression of STAT3, and melatonin administration remarkably reversed the breast cancer resistance to paclitaxel [91].

Due to the increased use of shorter wavelength illumination because of the advantages it provides, e.g., its energy-efficient properties, these lights have been reported to be associated with a variety of health problems, especially the progression of breast cancer [92]. Zubidat et al.[93] measured the urinary metabolite of melatonin (6-sulfatoxymelatonin), along with monitoring tumor growth and metastases, the status of global DNA methylation, and urinary levels of corticosterone in 4T1 breast cancer cells-tumor bearing female BALB/c mice; they observed ALAN-induced melatonin suppression by four different spectral light compositions (500–595 nm). The findings showed an inverted dose-dependent association between ALAN wavelength and suppression of melatonin. Short wavelength significantly caused an increase in tumor growth, the development of lung metastases, and extended hypomethylation of DNA, although long wavelength light has been reported to be responsible for lessening the mentioned effects. Also, melatonin administration caused a significant reduction of cancer burden. Their results suggested that through inducing aberrant methylation of DNA mediated by melatonin suppression, short wavelength light increases breast cancer burden. Moreover, global DNA methylation and suppression of melatonin secretion are suggested as promising early diagnosis and therapy biomarkers in breast cancer patients [93].

## Conclusion

**Fig. 1:**
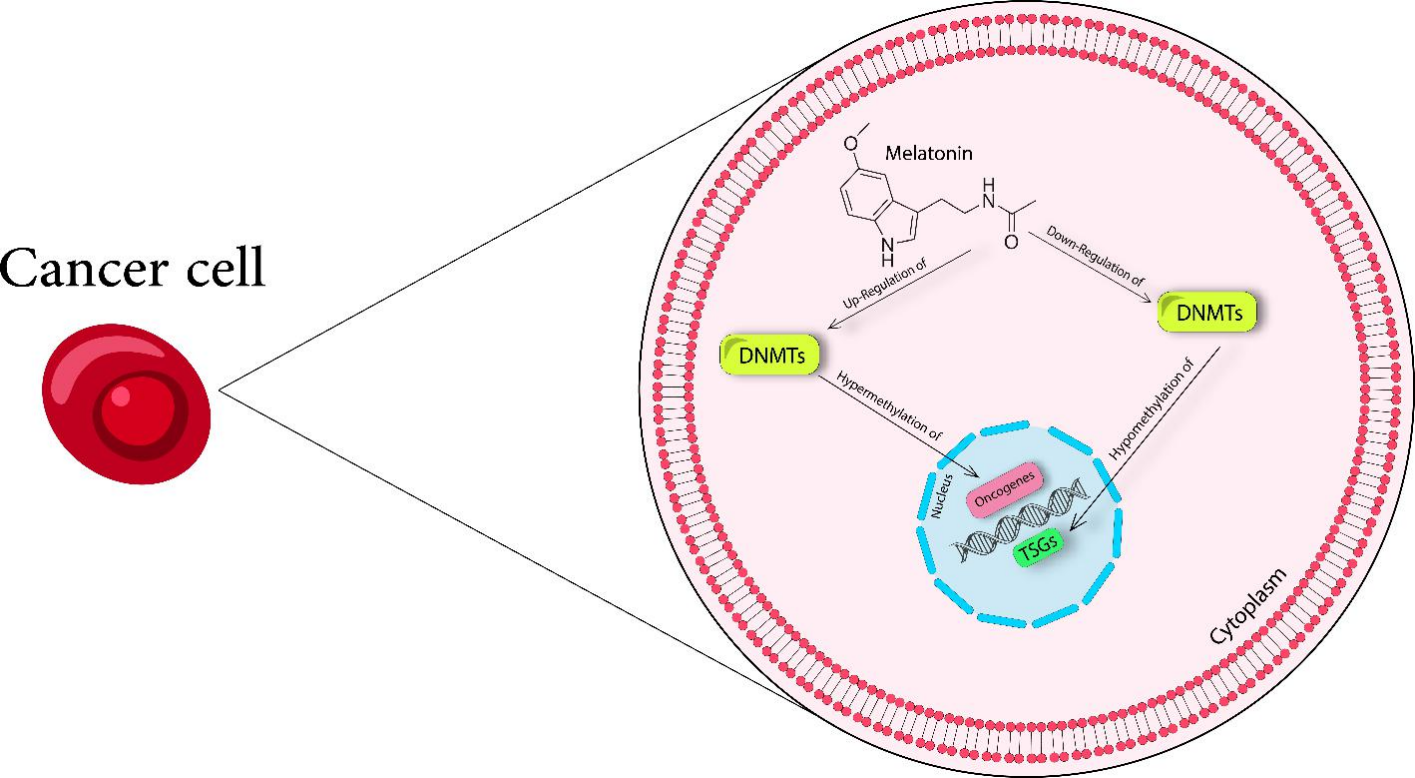
Melatonin and cancer suppression: Insights into its effects on DNA methylation

Currently, the utility of melatonin as an anti-cancer agent seems to be a promising and effective strategy for cancer management. One of the newest fields related to its anti-cancer properties is highlighted by its role in the regulation of numerous epigenetic processes including histone modifications, biogenesis of ncRNAs, and modification of DNA methylation. This summary reviewed the studies which investigated the properties of melatonin in the regulation of DNA methylation (Table 1 and Fig. 1). Indeed, we observed that through regulating the expression levels of distinct enzymes such as DNMTs, melatonin significantly modified the status of DNA methylation, especially in breast cancer tissue. Consequently, the changes in DNA methylation led to an inhibition of cancer cell proliferation, progression and metastasis, and reversed chemoresistance to current drug regimens by affecting several molecular pathways. Nevertheless, further well-designed mechanistic investigations are needed for a complete understanding of the molecular implications of melatonin-induced modification of DNA methylation against different cancer types, and the application of this promising agent as part of the chemotherapeutic regimen for patients in clinical practice.

**Table 1:** Studies that investigated the melatonin-induced modification of DNA methylation against cancer

Cancer Type	Cell line(s)	Study Model and Dosage(s)	Affected Gene(s)	Ref.
Malignant glioma	A172, U87, U373, and BTSCs	*In vitro*, 0–1mM for 24 and 48 h	ABCG2/BCRP	[77]
Colorectal cancer	Caco-2	*In vitro*, 1-100 nmol/L	NF-κB, IL‐6, -8, COX‐2, and NO	[79]
Breast cancer	4T1	*In vivo* (n = 12/group), 33 mg/L in drinking water	Global DNA methylation	[83]
Breast cancer	MCF-7	*In vitro*, 1 and 100 nM	EGR3, POU4F2/Brn-3b, and GPC3	[84]
Breast cancer	4T1	*In vivo*, 10 mg/L in drinking water	Global DNA methylation	[85]
Breast cancer	MCF-7	*In vitro* 10 nM for 24 h, and *In vivo* (n = 3/group) 0.1 mg/mL in drinking water	STAT3, IL-6, and ARH1	[90]
Breast cancer	4T1	*In vivo* (n = 10/group) 1.9 mg/kg/d	Global DNA methylation	[93]

## Data Availability

Not applicable.

## Abbreviations

ROSs: Reactive oxygen species
GI: Gastrointestinal
AANAT: Aralkylamine N-acetyltransferase
SCN: Suprachiasmatic nucleus
SCG: Superior Cervical Ganglia
CSF: Cerebrospinal Fluid
TNF-α: Tumor Necrosis Factor Alpha
IFN-γ: Interferon-gamma
IL-2: Interleukin-2
IL-4: Interleukin-4
IL-10: Interleukin-10
IL-27: Interleukin-27
MMPs: Matrix metalloproteinases
EMT: Epithelial-to-mesenchymal transition
QR2: Quinone reductase 2
cAMP: Cyclic adenosine monophosphate
PKA: Protein kinase A
cGMP: Cyclic guanosine monophosphate
CVDs: Cardiovascular diseases
T2DM: Type 2 diabetes mellitus
DNMTs: DNA methyltransferases
CRC: Colorectal cancer
TETs: Ten-eleven translocation enzymes
BTSCs: Brain Tumor Stem cells
DNMTi: DNA methyltransferase inhibitor
IL-6: Interleukin-6
IL‐8: Interleukin-8
COX‐2: Cyclooxygenase-2
NO: Nitric Oxide
NF‐κB: Nuclear Factor-κB
ALAN: Artificial Light at Night
IBD: Inflammatory Bowel Disease
qPCR: Real-time quantitative PCR
STAT3: Signal Transducer and Activator of Transcription 3
ARHI: Aplasia Ras Homolog one
dLAN: dim Light at Night
SIRT1: Sirtuin 1
